# Dysregulation of Connexin Expression Plays a Pivotal Role in Psoriasis

**DOI:** 10.3390/ijms22116060

**Published:** 2021-06-04

**Authors:** Erin M. O’Shaughnessy, William Duffy, Laura Garcia-Vega, Keith Hussey, A. David Burden, Mozheh Zamiri, Patricia E. Martin

**Affiliations:** 1 Department of Biological and Biomedical Sciences, School of Health and Life Sciences, Glasgow Caledonian University, Glasgow G4 0BA, UK; erinoshaughnessy@rocketmail.com (E.M.O.); v.lauramaria.garcia@gmail.com (L.G.-V.); 2 Department of Dermatology, University Hospital Crosshouse, Kilmarnock KA2 0BE, UK; william.duffy2@aapct.scot.nhs.uk (W.D.); mozheh.zamiri@glasgow.ac.uk (M.Z.); 3 Department of Vascular Surgery, Queen Elizabeth University Hospital, Glasgow G51 4TF, UK; keithhussey79@hotmail.com; 4 Institute of Infection Immunity and Inflammation, University of Glasgow, Glasgow G12 8TA, UK; David.Burden@glasgow.ac.uk; 5 Department of Dermatology, Queen Elizabeth University Hospital, Glasgow G51 4TF, UK

**Keywords:** psoriasis, epidermis, connexin26, connexin43, gap junction, hemichannels, connexin mimetic peptide

## Abstract

Background: Psoriasis, a chronic inflammatory disease affecting 2–3% of the population, is characterised by epidermal hyperplasia, a sustained pro-inflammatory immune response and is primarily a T-cell driven disease. Previous work determined that Connexin26 is upregulated in psoriatic tissue. This study extends these findings. Methods: Biopsies spanning psoriatic plaque (PP) and non-involved tissue (PN) were compared to normal controls (NN). RNA was isolated and subject to real-time PCR to determine gene expression profiles, including *GJB2/CX26*, *GJB6/CX30* and *GJA1/CX43*. Protein expression was assessed by immunohistochemistry. Keratinocytes and fibroblasts were isolated and used in 3D organotypic models. The pro-inflammatory status of fibroblasts and 3D cultures was assessed via ELISA and RnD cytokine arrays in the presence or absence of the connexin channel blocker Gap27. Results: Connexin26 expression is dramatically enhanced at both transcriptional and translational level in PP and PN tissue compared to NN (>100x). In contrast, CX43 gene expression is not affected, but the protein is post-translationally modified and accumulates in psoriatic tissue. Fibroblasts isolated from psoriatic patients had a higher inflammatory index than normal fibroblasts and drove normal keratinocytes to adopt a “psoriatic phenotype” in a 3D-organotypic model. Exposure of normal fibroblasts to the pro-inflammatory mediator peptidoglycan, isolated from *Staphylococcus aureus* enhanced cytokine release, an event protected by Gap27. Conclusion: dysregulation of the connexin26:43 expression profile in psoriatic tissue contributes to an imbalance of cellular events. Inhibition of connexin signalling reduces pro-inflammatory events and may hold therapeutic benefit.

## 1. Introduction

Psoriasis is a chronic inflammatory skin condition affecting 2–3% of the population, characterised by epidermal hyperproliferation triggered primarily by a T-cell mediated response, i.e., “inside-out” signalling events [1]. Environmental stresses, including a shift in the microbiome from a commensal to opportunistic phenotype (e.g., *Staphylococcus aureus*), may also play a role, i.e., “outside-in” signalling events [2,3,4,5,6]. Dysregulated epidermal hyperproliferation is associated with changes in the epidermal differentiation complex. Increased expression of the gap junction protein connexin26 (CX26), encoded by *GJB2/CX26*, was first reported in psoriatic tissue in the 1990s [7,8]. More recently, RNAseq analysis determined that *CX26* was within the top 100 genes upregulated in the psoriatic transcriptome [9], however, the role of CX26 in psoriasis and differential levels of expression within plaque border areas have not been studied in detail.

Connexins (CX) are a family of 21 highly conserved proteins that fall into discrete phylogenetic clusters with up to 10 subtypes expressed throughout the differentiated epidermis. Six connexins assemble to form normally closed hemichannels (HC) in the plasma membrane, followed by intercellular alignment and docking with neighbouring hemichannels to form intercellular gap junctions. These channels enable the exchange of metabolites and signalling molecules throughout the avascular epidermis. CX43, encoded by *GJA1/CX43*, predominates and is primarily associated with basal, proliferating layers. CX26 and CX30 are expressed at lower amounts and localise to spinous and granular layers in normal epidermis [10]. The connexins expressed from the *GJA* and *GJB* gene clusters are unable to form channels with each other and, thus, the epidermal GJ expression profile is believed to play an important role in maintaining epidermal integrity [11].

Dysregulation of connexin hemichannel signalling is a pathological trigger for a diverse range of disease states, including chronic non-healing skin wounds, retinopathies and neurodegenerative disorders ([12,13]); as such, connexin channel blockers emerge as prime therapeutic targets [12,13]. We recently reported that exposure to the pro-inflammatory mediator peptidoglycan (PGN), isolated from *S. aureus*, promotes CX26 expression in keratinocytes and ATP release, impacting downstream purinergic signalling pathways and inflammation [10,14]. These events can be attenuated by connexin channel inhibitors, including carbenoxolone, the connexin mimetic peptides (CMP) Gap27 targeted to the second extracellular loop of CX43 and by siRNA targeted to CX26 or CX43 in cell model systems [14,15,16].

In the present manuscript, we have uniquely used skin biopsies spanning psoriatic plaque border regions, enabling a comparative analysis of the connexin gene and protein expression patterns from clinically involved chronic psoriatic plaque (PP) and clinically non-involved psoriatic border regions (PN) to be correlated with normal tissue (NN). Three-dimensional (3D) organotypic models were also generated to gain further insight into epidermal connexin signalling. Cytokine arrays were used to assess the pro-inflammatory status of the 3D models grown on feeder layers of normal and psoriatic fibroblasts and the influence of PGN and Gap27 on pro-inflammatory events. The data are discussed in the context of diverse whole genome (WGA) and RNAseq analysis [17,18,19,20] and indicate that altered signalling via connexin channels is a key contributor to the psoriatic state.

## 2. Results

### 2.1. Epidermal Thickening in Perilesional and Lesional Tissue

**Figure 1:**
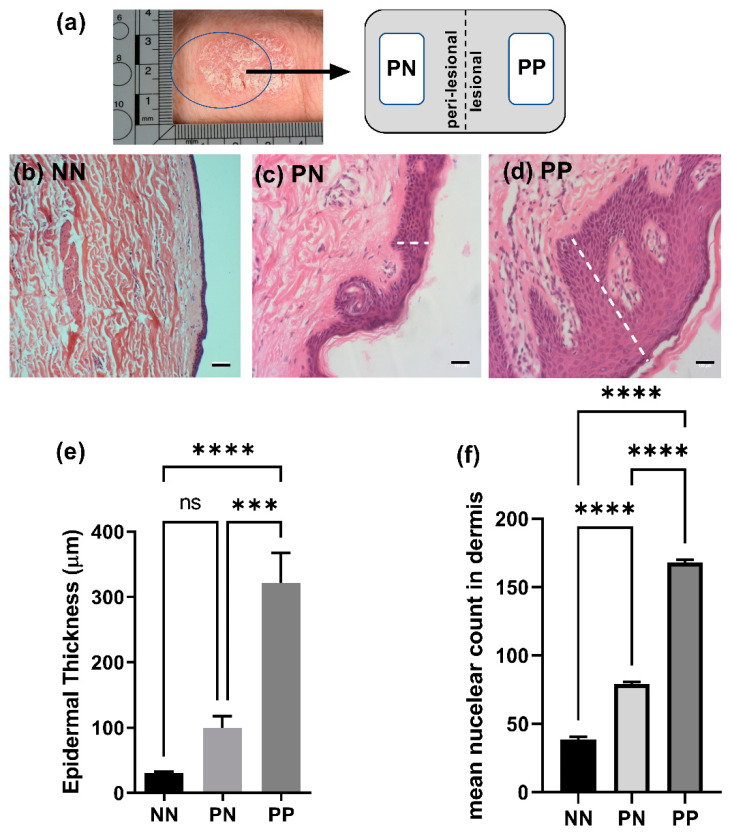
Histological analysis of psoriatic versus normal tissue biopsies. Tissue biopsies were collected from normal donors (NN), psoriatic non-involved (PN) or psoriatic plaque (PP) regions. (**a**) Diagrammatic representation of tissue biopsy sites from PP and PN locations; (**b**–**d**) representative images of NN, PN and PP biopsies are presented; dotted lines indicate examples of measurements taken; (**e**) epidermal thickness; (**f**) mean dermal nuclear infiltrate. *N* = 3 donors. Statistical significance was calculated by ANOVA followed by Tukey post-hoc test *** *p* < 0.005, **** *p* < 0.0001. Bar = 100 μm.

Biopsies from psoriatic patients and normal healthy donors were obtained following ethical consent (Section 4.1) and termed normal (NN), psoriatic plaque (PP) and psoriatic non-involved (PN) with tissue being within 1–1.5 cm from the plaque lesion border (Figure 1a). Histological analysis confirmed that the epidermis from normal donors (NN) was ~30 μm thick with ~5 discrete epidermal layers visible (Figure 1b,e). PN epidermis was ~three-fold thicker (~100 μm) with rete ridges beginning to elongate, although not statistically thicker than NN samples (Figure 1c,e). PP epidermis was at least ten-fold thicker than NN (~320 μm) and significantly thicker than PN tissue with classical histopathological features of psoriasis including elongation of rete ridges, acanthosis and parakeratotic hyperkeratosis evident (Figure 1d,e). Semi-quantitative analysis of nuclei staining in dermal layers confirmed enhanced inflammatory cell infiltrate in psoriatic tissue that was two-fold higher for PN and four-fold higher in PP regions than NN biopsies (Figure 1f).

### 2.2. Protein Expression in NN, PN and PP Tissue

**Figure 2:**
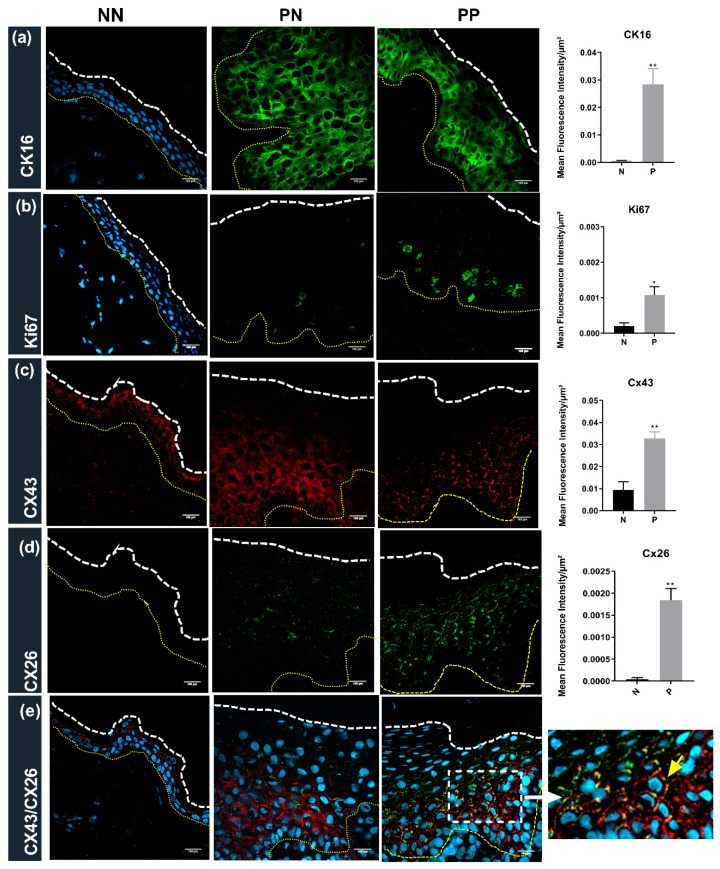
Immunohistochemistry of NN, PN and PP biopsies: frozen sections of biopsies were sectioned (7 um) and subject to immunohistochemical analysis. Representative images for (**a**) CK16 (green); (**b**) Ki67 (green); (**c**) CX43 (red) and (**d**) CX26 (green) are presented; (**e**) merged images of CX43, CX26 and DAPI (blue). The right-hand panel magnified insert with the yellow arrow indicating co-localisation areas. The white dotted line indicates tissue edge, and yellow dots indicate basement membrane. Bar = 100 μm. The mean fluorescent intensity/μm^2^ extracted from multiple images of three donors for each group is indicated in the right-hand panel for each marker. Statistical significance was calculated by two-tailed *t*-test * *p* < 0.05; ** *p* < 0.01.

Cytokeratin 16 (CK16), a hallmark of psoriasis [21,22,23], was barely detectable in normal epidermal tissue (Figure 2a) and was significantly upregulated in the cytoplasm of PN and PP biopsies (Figure 2a). Ki67, a marker for cell proliferation [24], was also barely evident in NN tissue (Figure 2b), with proliferation significantly upregulated in basal layers of PN and PP biopsies (Figure 2b). CX43 was widely expressed throughout the normal epidermis, especially in the spinous layer (Figure 2c). The level of CX43 was significantly increased in psoriatic tissue (Figure 2c), particularly in the spinous layer of the PN sections (Figure 2c), where intracellular staining was evident compared to the PP regions where the protein predominantly localised to the plasma membrane (Figure 2c and in the magnified images in Figure 4c,f,i). CX26 is not normally expressed at high levels in the normal epidermis, as confirmed in Figure 2d. In contrast, CX26 was expressed throughout the psoriatic epidermal tissue (PN or PP) (Figure 2d). The expanded epidermis in psoriasis was also evident by co-staining with DAPI in each section, as illustrated in Figure 2e. The co-localisation of CX43 and CX26 was also evidenced in the expanded spinous layer, clearly seen in the magnified insert panel of the PP tissue, with less CX26 present in basal areas (Figure 2e). The mean fluorescent intensity (MFI) of each marker (Figure 2a–d) is represented in the right-hand panel, confirming the profound upregulation of protein expression in psoriatic tissue compared to normal controls.

### 2.3. Gene Expression in NN, PN and PP Tissue

**Figure 3:**
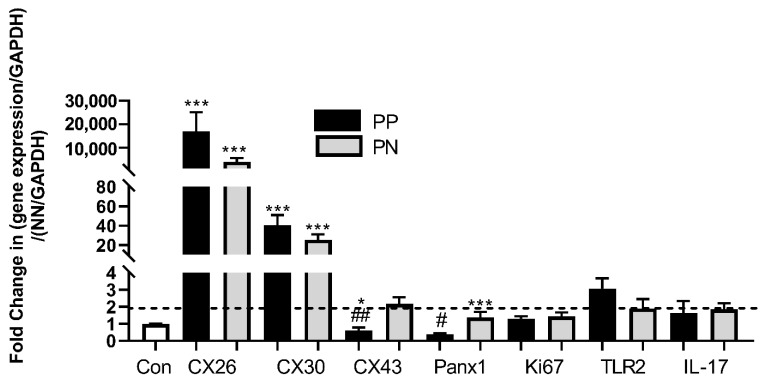
Gene expression profiles of NN, PN and PP biopsies. RNA was extracted from NN, PN and PP tissue biopsies and subject to RT-PCR analysis. Results are presented as the fold change in gene expression/GAPDH compared to NN gene expression/GAPDH. Data sets were statistically analysed by ANOVA followed by Dunnett’s multiple comparison test comparing PP and PN samples with control (***) and PN with PP samples (##). *N* = 6 biopsies per patient *** *p* < 0.0001; ^##^ *p* < 0.005; * ^(#)^ *p* < 0.01. The dotted line indicates a two-fold increase in gene expression considered biologically significant [25].

Expression of a panel of genes associated with connexin signalling and/or psoriasis was assessed by real-time polymerase chain reaction (RT-PCR) of NN, PN and PP tissue (Figure 3). Expression of *CX26* was dramatically upregulated in tissue from PP regions where gene expression was up to 20,000-fold higher than normal tissue (Figure 3). In PN regions, *CX26* expression was significantly greater than NN tissue (~5000-fold) (Figure 3) with a similar trend for *CX30* where expression was ~30-fold greater than NN tissue (Figure 3). There was no significant change in the expression of *CX43* (Figure 3) in PP biopsies compared to NN. However, in samples taken from PN regions, *CX43* expression was enhanced two-fold (Figure 3). No biologically significant changes in *Panx1* gene expression were observed, although the level of expression in PP regions was ~two-fold less than that in PN areas (Figure 3). The level of gene expression of Ki67 in PP or PN tissue was no different to that of normal tissue, while the level of TLR2 and IL-17 expression was ~2.5-fold higher in PP biopsies (Figure 3). Thus, from the panel of genes examined *CX26* and *CX30* were consistently upregulated in psoriatic tissue, although individual levels of expression between patient samples were highly variable, with some patients being “superexpressors” of *CX26*.

### 2.4. *CX43* Is Post-Translationally Modified in Psoriatic Epidermis

**Figure 4:**
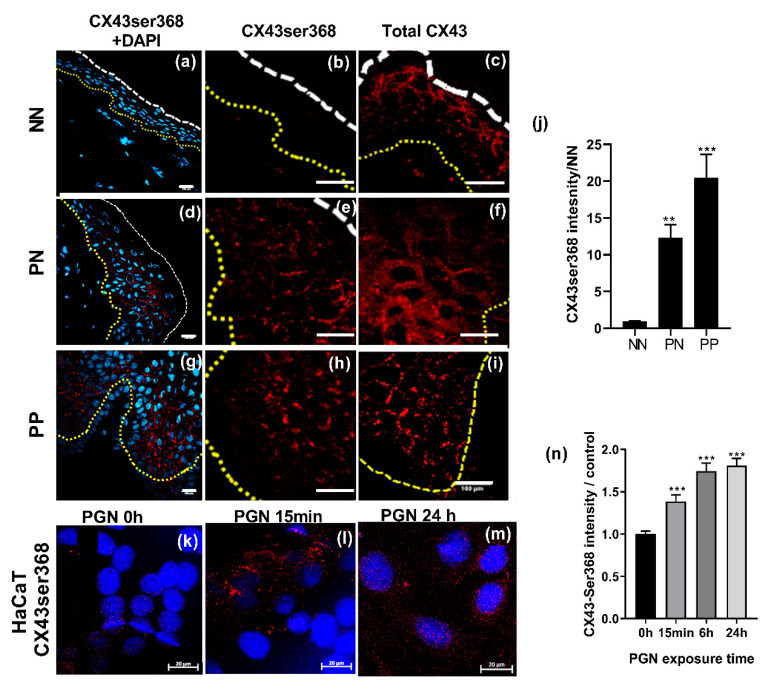
CX43 is post-translationally phosphorylated in psoriatic tissue and following the PGN challenge of keratinocytes. Frozen sections of biopsies were sectioned and stained with an antibody targeted to phosphorylated CX43ser368 (red and DAPI blue) or an antibody targeting total CX43. Representative images of NN (**a**–**c**); PN (**d**–**f**) and; PP (**g**–**i**) are presented. Bar = 100 μm; (**j**) The fold increase in mean fluorescent intensity (MFI) extracted from multiple images of CX43ser368 staining of PN and PP biopsies over NN is represented (*N* = 4 donors). HaCaT cells were exposed to 10 μg/mL PGN for 15 min to 24 h followed by fixation and staining for CX43ser368 (**k**–**m**) bar = 20 μm. The MFI extracted from multiple images of CX43ser368 staining in PGN treated samples/control is represented in (**n**) (*N* = 3). Data were statistically analysed by one-way ANOVA followed by Dunnet’s multiple comparison analysis against relevant control (NN or 0 h) ** *p* < 0.005; *** *p* < 0.0001).

The data from the gene and protein expression of total CX43 suggested that upregulation of CX43 was possibly due to a stall in the CX43 life cycle or a post-translational event [26]. The level of CX43ser368 phosphorylation in the normal epidermis was barely detectable, with some isolated areas of punctate staining observed (Figure 4a,b). This is in contrast to the extensive staining of total CX43 observed in the NN tissue (Figure 2c) (NN) and in the magnified NN image of total CX43 staining (Figure 4c). By contrast, in PN and PP biopsies, high levels of CX43ser368 phosphorylation was observed (Figure 4d,e,g,h). In the PN biopsies, staining was mostly localised to the plasma membrane areas (Figure 4e) compared to the intracellular staining observed using an antibody detecting total CX43 (Figure 4f). In PP biopsies, both CX43ser368 and total CX43 staining was predominantly located at plasma membrane regions indicative of gap junction plaque formation (Figure 4g–i) Semi-quantitative analysis, expressed as the mean fluorescence intensity (MFI), from four psoriasis patients and two controls confirmed the observed upregulation (Figure 4j).

### 2.5. *CX43* Is Post-Translationally Modified in Keratinocytes Exposed to the Pro-Inflammatory Mediator Peptidoglycan Isolated from S. aureus

Previously we determined that Cx-HC signalling was triggered by exposing keratinocytes to peptidoglycan (PGN), stimulating downstream signalling events including the expression of IL-6, IL-8 and CX26, and on prolonged exposure, decreased CX43 protein expression [14,15,16]. This data suggests that an “outside-in” signalling route can alter connexin expression profiles and function. Under basal conditions, limited phosphorylation of CX43 was evident in HaCaT cells, a model human keratinocyte cell line (Figure 4k). However, exposure of HaCaT cells to 10 μg/mL PGN for 15 min to 24 hours (h), induced CX43ser368 phosphorylation (Figure 4k–n), further suggesting that pro-inflammatory events and an “outside-in trigger” can alter CX43 phosphorylation status.

### 2.6. A 3D Organotypic Model of Psoriatic Epidermis

**Figure 5:**
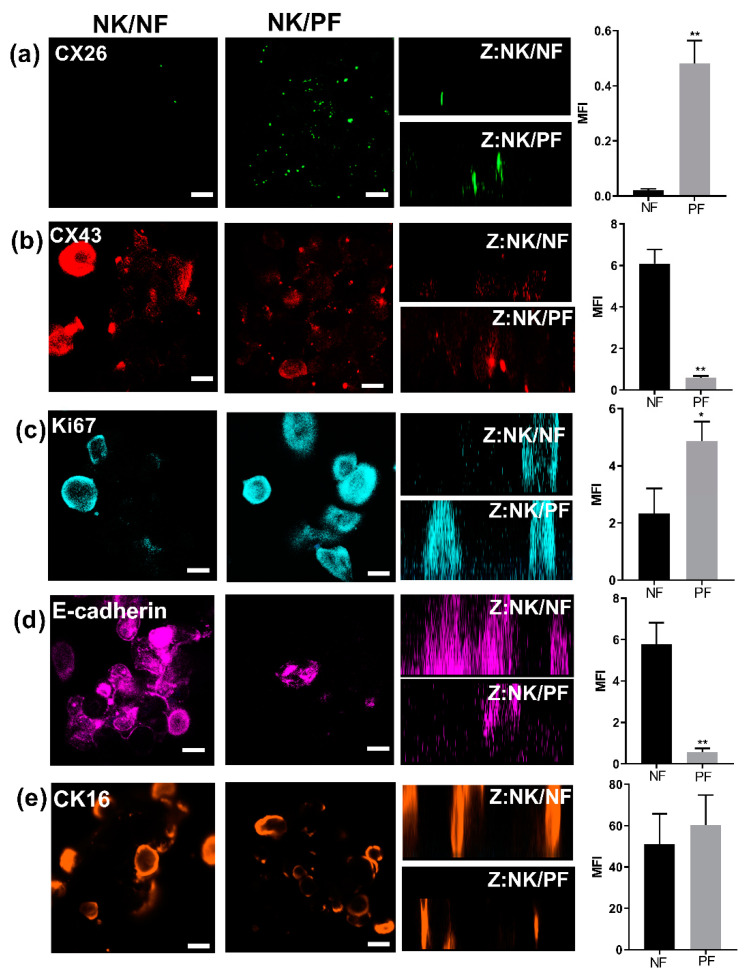
3D Organotypic epidermal model. Keratinocytes grown in transwells on either a normal (column NK/NF) or on a psoriatic (column NK/PF) fibroblast feeder layer were fixed and stained after 12 days at the ALI with relevant antibodies followed by 3D z-stack reconstruction (Z: NK/NF). IHC analysis determined expression of (**a**) CX26, (**b**) CX43, (**c**) Ki67, (**d**) E-cadherin and (**e**) CK16 protein expression. (**a**–**e**) bar = 100 μm. The mean fluorescent intensity of multiple regions from different cultures was extracted (right hand panel). Statistical analysis was performed by Student *t*-test *N* = 3. * *p* < 0.05; ** *p* < 0.001.

**Figure 6:**
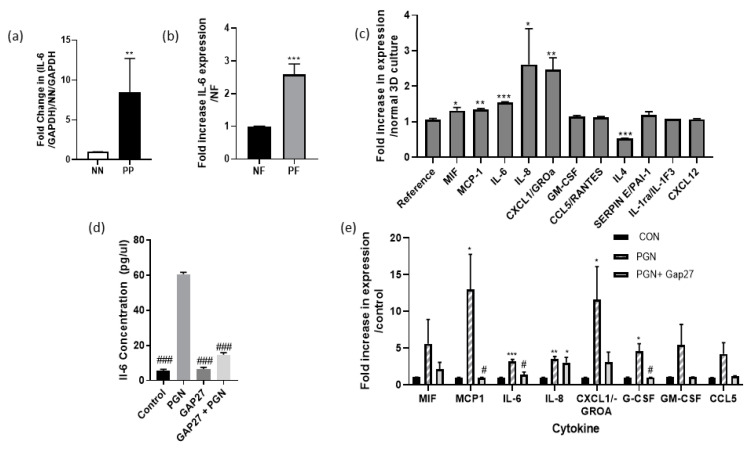
Psoriatic fibroblasts have an increased pro-inflammatory profile that can be reduced by connexin mimetic peptide Gap27. (**a**) RNA was extracted from NN and PP tissue biopsies and subject to RT-PCR analysis to assess IL-6 gene expression (*N* = 6 biopsies per patient); data are expressed as the fold change in IL-6 expression compared to normal tissue. (**b**) Media was collected from NF and PF cells and subject to ELISA assay for IL-6 (*N* = 6); data are expressed as the fold increase in IL-6 protein expression compared to NF. (**c**) Following 12 days at the ALI and generation of the 3D organotypic model the media was collected and subject to a RnD cytokine array. The relative change in cytokine expression PF vs. NF cultures was compared (*N* = 3). Statistical analysis was performed via two-tailed *t*-test to determine significance compared to NN or NF samples (**a**,**b**) or to the reference controls on the arrays that were unaltered between samples (**c**). * *p* < 0.05, ** *p* < 0.001; *** *p* < 0.0001. (**d**,**e**) NF were subject to 24 h exposure to 10 μg/mL PGN in the presence or absence of 100 nM Gap27 and the collected media was subject to (**d**) IL-6 ELISA assay (*N* = 6) or (**e**) to a R&D cytokine array where the fold change in cytokine protein expression over control was determined (*N* = 4). One-way ANOVA followed by Dunnett’s multiple comparison determined the significant differences between (**d**) PGN challenged with control and PGN+Gap27 ^#^ *p* < 0.05, ^###^ *p* < 0.001 and (**e**) within each cytokine group to the control * *p* < 0.05, ** *p* < 0.001; *** *p* < 0.005.

The PGN model provides an example of an “outside-in” trigger and reflects the changes in CX expression that occurs in the psoriatic epidermis. The functional consequences contribute to purinergic signalling pathways that also play an important role in psoriasis [14,27]. To explore these events, further 2D and 3D organotypic models utilising primary keratinocytes and fibroblasts grown on transparent membranes were used [28,29]. In contrast to the isolation and culture of keratinocytes from normal and diabetic donors that have been successfully used previously in this approach [29], keratinocytes derived from psoriatic patients were difficult to passage following initial isolation. Therefore, 3D organotypic models explored the impact of fibroblasts isolated from PP tissue biopsies (psoriatic fibroblasts (PF)) on the stratification of normal adult primary keratinocytes (NK) in comparison to those grown on normal fibroblast (NF) feeder layers (Figure 5a–e). In psoriatic patient skin biopsies, RT-PCR determined that IL-6 expression levels were higher in PP biopsies than that from NN donors (Figure 6a). Following isolation and culture of NF and PF cells, the media was harvested and IL-6-ELISA assays determined that the psoriatic fibroblasts had a higher pro-inflammatory status than normal adult fibroblasts (Figure 6b). Thus, we hypothesised that this “inside-out” pro-inflammatory status would drive an increase in CX26 expression and other changes seen in the epidermis of psoriatic tissue. Following three weeks of culture at the air-liquid interface (ALI), cells were fixed and keratinocytes stained in situ for CX26, CX43, Ki67, E-cadherin and CK16. When grown on an AF feeder layer, CX26 was present in very low amounts, which was clearly increased in the models grown on the PF feeder layer (Figure 5a). CX43 was readily detected in both normal and psoriatic models, with evidence of CX43 expression in stratified regions in the psoriatic model (Figure 5b). Increased Ki67 staining was observed in the psoriatic model (Figure 5c); CK16 was equally expressed in both, while the level of E-cadherin was reduced in the psoriatic model (Figure 5d,e). Thus, the model reflected the expression profile of many aspects of the psoriatic epidermis, suggesting that soluble/diffusable factors released by psoriatic fibroblasts can drive alterations in keratinocyte status.

Upon termination of the experiment, the media from the 3D cultures was harvested and subject to a RnD cytokine array. The data from the arrays were expressed as the fold increase in cytokine expression in the NK/PF culture to the NK/NF cultures. In agreement with the initial ELISA assay, at the start of the experiment, IL-6 expression was significantly higher in the psoriatic model. Increased expression of IL-8, chemokine (C-X-C motif) ligand 1(CXCL1), macrophage migration inhibitory factor (MIF) or monocyte chemoattractant protein 1(MCP-1) were also noted (Figure 6c). Thereby indicating that the enhanced inflammatory status of the PF and “inside-out” signalling events were involved in promoting the changes seen in the 3D organotypic epidermis.

### 2.7. The Connexin Mimetic Peptide GAP27 Reduces PGN Evoked Pro-Inflammatory Responses in Adult Fibroblasts

To determine if PGN challenge evoked similar trends in cytokine expression seen in the 3D “psoriatic model” 2D monocultures of NF were challenged with 10 μg/mL PGN for 24 h. An ELISA assay determined that IL-6 expression was clearly induced following challenge (Figure 6d) and RnD cytokine arrays determined that expression of a range of cytokines including MIF, MCP-1, IL-6, IL-8, CXCL1, granulocyte colony-stimulating factor (GCSF), granulocyte-macrophage colony-stimulating factor (GMCSF) and chemokine ligand 5 (CCL5) were induced (Figure 6e). Previously we have shown that PGN challenge also evoked connexin hemichannel activity and that this was related to an enhanced cytokine response that can be inhibited by connexin channel blockers [14,15,16]. The connexin mimetic peptide Gap27 has also previously been shown to inhibit Cx hemichannel activity and enhance wound closure rates in normal adult fibroblasts and keratinocytes [30]. Exposure of NF to Gap27 on its own had no effect on IL-6 expression (Figure 6d), however, it significantly reduced the PGN evoked IL-6 response (Figure 6d). When applied to the cytokine arrays, no significant induction of cytokine expression was seen in supernatants from cells exposed to PGN and 100 nM Gap27 (Figure 6c).

## 3. Discussion

In the present study, we report for the first time a detailed comparison of the gene and protein expression profiles of CX26 and CX43 in NN, PN and PP tissue biopsies. We conclude that enhanced CX26 is a key molecular footprint of psoriasis and that upregulation of CX26 expression and function critically relate to pathophysiological pathways that trigger the condition.

Within the normal epidermis, the levels of CX26 expression are barely detectable by immunohistochemical staining, however, in hyperproliferative skin, CX26 levels are enhanced. This was originally reported in the 1990s in psoriatic tissue, human papillomavirus infected warts and at the wound edge of chronic non-healing wounds [7,8,31]. In the present study, *CX26* gene expression was enhanced in all biopsies taken from psoriatic patients compared to normal skin biopsies, with significant variation ranging from 5 to >1000-fold over normal tissue, indicating patient variability. Extraction of data from multiple mRNAseq and WGA from PP/PN/NN confirm that *CX26* is consistently enhanced. In an initial mRNAseq analysis, *CX26* was in the top 100 genes upregulated with an 18-fold increase over normal tissue, *CX30* expression was 7-fold higher, however, no changes in expression of *CX43* were observed [9]. Psoriasis is a multifactorial disorder with a diverse array of triggers with different forms associated with distinctive areas of the body. All biopsies in the present study were collected from patients with chronic plaque psoriasis (CP), with tissue isolated from trunk and pressure point areas. In a study by Ahn et al., differential gene expression and ingenuity pathway analysis performed on biopsies isolated from chronic plaque (CP), scalp psoriasis (SP) or palmoplantar psoriasis (HF) [17] revealed that *CX26* and *CX30* were enhanced in CP and SP compared to NN tissue, in line with the present study. By contrast, in HF, only *CX30* was consistently upregulated. Studies of Chinese populations have suggested that *CX30* was a suitable biomarker for psoriasis [32,33,34], and in WGA of Chinese populations, *CX30*, but not *CX26* expression, was upregulated [19,20]. In our study, in patients where *CX26* levels were not excessively upregulated, the levels of *CX30* expression tended to be higher; the relevance of this warrants further analysis in a broader patient cohort. In the present study, *CX26* and *CX30* were the only markers analysed that were consistently upregulated in all of the patient biopsies analysed.

CX26 assembles to form HC in the plasma membrane, which can release ATP into the extracellular space prior to aligning with neighbouring hemichannels to form gap junction units [11]. It is well established that aberrant CX26 expression and function is associated with epidermal dysplasia [35]. In a mouse model of Cx26 overexpression, where Cx26 was under the control of the involucrin promoter, mice developed a psoriasiform phenotype with enhanced proliferation, inflammation and delayed wound closure response [36]. In another model, the application of a tumour promoting agent evoked Cx26 expression [37]. Tape stripping of the normal human epidermis also induced an increase in CX26 prior to the onset of hyperproliferation, assessed by Ki67 staining, suggesting epidermal trauma can evoke increases in CX26 expression and may relate to HC sensitivity to mechanical stimulation [8,38]. Mutations in CX26 are associated with a wide range of epidermal disorders of differing severity, including keratitis ichthyosis deafness syndrome, an inflammatory skin disorder with susceptibility to opportunistic skin infections and associated with “leaky” CX26 hemichannels and altered oligomerisation profiles [11,39,40,41].

In single-cell mRNAseq analysis from PP and NN tissue, Cheng et al. captured keratinocytes by laser dissection from discrete layers of the epidermis and analysed PP/NN expression ratios in ingenuity pathways [42]. The data was extracted as the log odds of differential gene expression between PP/NN keratinocytes that were classified into discrete units basal, follicular, channel, mitotic, spinous, etc. The odds of *CX26* and *CX30* upregulation in basal, mitotic and spinous layers was ~1000-fold. In a population of keratinocytes enriched with channel markers the likelihood of *CX26* and *CX30* upregulation was 7–8000 but *CX43* or *CX45*(*GJA5*) were unlikely to be altered.

The core psoriatic pathways identified by Ahn et al. [18] included MIF innate, purine nucleotide degradation and inflammasome activation. Overexpression and activation of CX26 feed into each of these pathways. Although HC are normally closed, environmental triggers including hypoxia and microbiome alterations open HC triggering pro-inflammatory mediated pathways and inhibitors of HC function emerge as key therapeutic targets for diverse inflammatory conditions [43,44,45,46,47]. Human CX26-HCs are predicted to be in a constitutively open state [48]. In normal skin this may be critical where ATP release is known to be a driver for normal cellular differentiation and calcium wave propagation [38,49,50]. Upregulation of CX26, leading to both an increase in CX26-HC in the plasma membrane and their ability to laterally accrete and dock to form Gap junctions would also provide more CX26-HC sensitivity to mechanical stimulation [38], causing an imbalance in epidermal signalling through the avascular epidermal layers. This would result in increased ATP release from cells, feeding into purinergic signalling pathways, a key trigger for psoriasis and associated with activation of pro-inflammatory events and altered differentiation pathways in the epidermis [27,51,52]. Recently we dissected these events in HaCaT cells where PGN exposure triggered CX26 expression, HC activity, monitored by ATP release and IL-6/IL-8 expression, events that were all controlled by inhibition of CX26 channel function, NfkB and purinergic inhibitors [14]. The presence of enhanced levels of ATP released from cells via CX26 channels would enter purine nucleotide degradation pathways. In the present study, we showed that the connexin mimetic peptide GAP27 that primarily inhibits Cx43 signalling reduced PGN-evoked cytokine release in adult fibroblasts that predominantly express CX43, further supporting strategies that target connexins’ therapeutic potential for chronic inflammatory conditions [43]. Antisense technologies targeting both Cx43 and Cx26 have reported benefits in in vivo wound healing studies, and a recent report by Becker and colleagues suggests that preventing upregulation of CX26 reduces inflammation and epidermal thickening [53]. Further studies are now warranted to explore if targeted inhibition of CX26 and CX30 in keratinocytes hold therapeutic benefit for psoriasis. No change in expression of *Panx1* was observed in the psoriatic biopsies and we previously determined that PGN exposure did not alter Panx1 protein expression levels in keratinocytes [14]. However, the activation of Panx1 channels cannot be ruled out, particularly as activation of Panx1 is closely related to triggering the inflammasome [54,55], a pathway identified by Ahn et al. to be central to psoriasis [17].

By contrast to the change in *CX26/CX30* gene expression, no change in *CX43* gene expression was observed in the present work or in available WGA and mRNAseq data. Furthermore, in the study by Cheng et al. on keratinocyte populations, *CX43* had a low prediction score (<30) in the channel subpopulation of keratinocytes [42] suggesting other roles for CX43. We identified that CX43 protein expression was significantly upregulated in psoriatic epidermal layers and that this was associated with phosphorylation of CX43 at position ser368 on the carboxyl tail. Several studies suggest that a chronic inflammatory environment can alter CX43 expression. In chronic non-healing, epithelial CX43 is dramatically upregulated at the wound edge, where both Cx mimetic peptides and SiRNA approaches show therapeutic potential in improving wound healing and resolving inflammation [30,47,56,57]. In endothelial cells, exposure to PGN rapidly evoked Cx43ser368 phosphorylation, followed by a decrease in protein expression [15]. Similarly, in HaCaT cell models, CX43 expression was reduced following 24 h exposure to PGN with Cx43ser368 phosphorylation and redistribution of the protein to intracellular stores and perinuclear regions evident in the present work [14]. In other recent studies, exposure of HaCaT cells to IL-22 resulted in the loss of gap junction coupling and a decrease in CX43 expression, although no changes in CX30 or CX26 were reported [58]. The decrease in CX43 expression was linked to the JNK pathway but not NfKB. In contrast, in the study by Garcia-Vega et al., the inhibition of NfKB was clearly linked to a decrease in PGN activated CX26 expression and function [14], recently reviewed by [10]. While promoter regions of *CX26* have NfkB regulatory domains, these have not been associated with *CX43* gene expression. In other studies, increased IL-6 expression in systemic inflammation has been linked with the downregulation of Cx43 expression in diverse tissues [59].

The rapid life cycle of Cx43 undergoes cyclic changes in phosphorylation profiles. The phosphorylation status of Cx43 is critical for the removal and targeted degradation of Cx43 [26,60]. Cx43 is rapidly removed from junctional areas upon phosphorylation which occurs on multiple serine residues on the carboxyl-terminal tail driven by PKC, MAP and ERK and PKA mediated events. Phosphorylated Cx43 is also targeted for ubiquitin degradation pathways, a major pathway upregulated in CP psoriasis but not associated with SP or HF [18]. Thus, we propose that phosphorylated CX43 is targeted for degradation in psoriasis, which may also explain the differences in the intracellular localisation of the protein in PP biopsies and HaCaT cells following prolonged exposure to PGN.

In the present study, we had difficulty in expanding keratinocyte cultures isolated from psoriatic tissue compared to standard procedures used for NN keratinocytes. In contrast, fibroblasts were readily sourced. Fibroblasts from psoriatic patients displayed a higher pro-inflammatory index than those isolated from normal patients with markers including IL-6, IL-8 and MIF upregulated, all of which were identified to be upregulated to different degrees in psoriatic tissue [17,18]. 3D organotypic models of normal keratinocytes grown on PF feeder layers developed an epidermal profile similar to that observed and reported in psoriatic tissue. CX26 and Ki67 levels were increased with a decrease in E-cadherin protein expression, suggesting this model could be further developed to dissect the impact of drugs targeting connexin related pathways and environmental triggers on patho-physiological mechanisms leading to psoriasis [61]. In a study on therapeutic modulators in psoriasis, Shaker and colleagues assessed CX26 expression in biopsies of patients before and after the classical psoriatic therapies—methotrexate and PUVA [62]. In all the biopsies, CX26 was enhanced in non-treated psoriatic tissue, with follow-up biopsies revealing decreased levels of CX26 clearly linked to clinical improvement. It remains to be determined if the diverse changes in *CX26* gene expression plays a part in psoriasis disease severity, and whether it is triggered by the Koebner phenomenon [63] or at mechanical stretch points, recently suggested to trigger stimulated keratinocyte proliferation and cytokine release [64].

In conclusion, maintaining the balance of CX26:CX43 expression and function is critical for maintaining epidermal integrity. It is evident that therapeutic pathways controlling CX26:43 expression and channel activity hold potential. For example, current drugs in use targeting NfKB pathways may play a role in reducing *CX26* thereby decreasing aberrant CX26 signalling helping resolve the inflammatory status that trigger hyperproliferation.

## 4. Materials and Methods

### 4.1. Biopsy Recruitment

Incisional elliptical skin biopsies (approximately 2.5 cm thick and 3 cm long) were collected from 19 psoriatic patients following ethical consent in the Department of Dermatology, University Hospital Crosshouse (ethical approval REF-16WS-0024, (age range 23–76)). Patients were not undergoing biologic treatments and no topical treatment was applied to the biopsy area for 7 days prior to the biopsy being taken (Figure 1a). Biopsies were termed psoriatic plaque (PP) and non-involved (PN) with tissue being within 1–1.5 cm from the plaque border. Control tissue (NN) from normal healthy donors was acquired from the GCU Skin Tissue bank (REF 16ES-0069). Tissue was collected in Dulbecco’s Modified Eagle’s Medium (DMEM, SLS, Newhouse, UK).

### 4.2. Cell Culture and 3D Organotypic Model

Fibroblasts and keratinocytes were isolated from tissue biopsies as previously described [30,65]. Briefly, subcutaneous fat was removed from tissue biopsies and tissue was exposed to dispase (ThermoFisher, Paisley, UK) (5% *w*/*v*) overnight at 4 °C to separate epidermal and dermal layers. Separated pieces of dermis were cut into approximately 1 × 1 mm^2^ pieces. Explanted tissue was placed in 6 well plates and overlaid with DMEM supplemented with 10% foetal calf serum, L-glutamine (2 mM) and 100 μg/mL) penicillin/streptomycin (cDMEM) (Lonza, Slough, UK), cultured at 37 °C/5%CO_2_ for 48–72 h to adhere. Media was changed every 2–3 days until cells reached confluence when they were washed in PBS, detached using TrypLe (Invitrogen, Paisley, UK) dissociating agent and expanded in T75 flasks.

Fibroblasts and HaCaT cells, a model human keratinocyte cell line (CLS, Eppelheim, Germany [66]), were cultured in cDMEM. Fibroblasts were banked at passage 1–2 or used for up to 4 passages. Keratinocytes derived from biopsies were cultured in Epilfe-S7 Media (Fisher Scientific UK Ltd., Loughborough, UK) and were used up to 4 passages (NK).

For the 3D organotypic model, normal (NF) or psoriatic fibroblasts (PF), isolated from psoriatic plaque (PP) areas, were grown to confluency on 6-well plates. Normal keratinocytes (NK) (1 × 10^6^ cells) were seeded onto polyester transparent transwell inserts (ThermoFisher, Paisley, UK) in EplifeS7 and media was changed every 2 days until confluency. The insert was raised to the ALI for 12 days to enable keratinocyte stratification with 600 μL media maintained in the wells beneath the transwell at all times [28,65,67].

### 4.3. Cell Challenges

HaCaT cells and NF were challenged with 10 μg/mL peptidoglycan (PGN) isolated from *S. aureus* (Sigma-Aldrich, Irvine, UK) for 15 min to 24 h prior to harvesting media and fixation of cells [14]. Where appropriate, cells were exposed to the connexin mimetic peptide Gap27 (100 nM (Zealand Pharma, Glostrup, Denmark)) throughout the PGN challenge [14].

### 4.4. Histological Analysis

The tissue (1–2 mm^2^) was fixed in paraformaldehyde 4% (*w*/*v*) overnight at 4 °C, wax embedded and stained by H&E following routine procedures. Slides were viewed under an Axiovert 200 inverted microscope linked up to a Zeiss digital 100 camera and images processed using ZEN 2. Blue edition software (Carl Zeiss Microscopy GmbH, Jena, Germany) and Image J.

### 4.5. Immunohistochemistry

Tissue taken directly from the DMEM media (~0.25 mm^2^) was mounted in optimal cutting temperature compound (OCT) (ThermoFisher, Paisley, UK) and 7 μm sections cut onto pre-coated slides. Tissue sections, organotypic cell models or cells cultured on 16 mm^2^ glass coverslips were fixed in ice-cold methanol prior to permeabilisation and blocking, as previously described [14]. The primary antibodies: rabbit polyclonal anti-Cx43 (1:100 dilution, [68]); mouse monoclonal anti-Cx26 (1:50 dilution, 13–8100, ThermoFisher, Paisley, UK); rabbit polyclonal anti-Ki67 (1:500, Abcam, Cambridge, UK, ab15580); rabbit polyclonal anti-cytokeratin16 (1:100; Abcam, Cambridge, UK ab53117); rabbit polyclonal anti-Cx43^pSer368^ (1:100; Sigma-Aldrich, Irvine, UK) and relevant secondary antibodies (goat anti-mouse Alexa 488 or -rabbit Alexa 594 (1:500 dilution; ThermoFisher, Paisley, UK) were used. Nuclei were counterstained with DAPI (2.5 μg/mL) (Sigma-Aldrich, Irvine, UK). Samples were visualised on a Zeiss LSM 800 confocal microscope. The mean fluorescence intensity/μm^2^ (MFI) was extracted for repeat areas of each image captured using Zen 2. Blue edition software (Carl Zeiss Microscopy GmbH, Jena, Germany).

### 4.6. RNA Extraction and Real Time PCR Analysis

Tissue (0.5 mm^2^) preserved in RNA Later (Life Technologies, Paisley, UK) was cut into small pieces and added to re-enforced extraction vials containing 450 μL of Promega ™RNA lysis buffer (Promega, Southhampton, UK) prior to homogenisation using a bead-beater. Tissue debris was collected following microfugation and RNA extracted using the Nucleospin RNA 11 extraction kit (Bioline, London, UK) according to the manufacturer’s instructions. Purified RNA was diluted to 350–400 ng/µL and cDNA was synthesised as previously described [14]. Gene expression was quantified by Taqman real-time polymerase chain reaction (RT-qPCR) using primers (sourced from IDT, Leuven, Belgium) targeting the following genes: *CX26; CX30; CX43; Panx1*, *IL-6; IL17; TRL2; Ki67;* and *GAPDH* (Supplementary Table S1). The cycle threshold value (Ct value) was extracted and normalised against the Ct of GAPDH (ΔΔCt method) [69], where ΔCt = Ct target gene − Ct GAPDH, ΔΔCt = ΔCt psoriatic tissue − ΔCt normal tissue, Gene expression = 2^−ΔΔCt^. Data is represented as the fold-increase of the mean value of the psoriatic tissue over the control tissue. Gene fold changes ≥ ±2 were considered significant [25,69].

### 4.7. ELISA Assays and Cytokine Array

Supernatants were collected from NF and PF fibroblasts at passage 2, HaCaT cells and NF before and after 24 h challenge with 10 μg/mL PGN. Samples were centrifuged for 5 min, transferred to fresh tubes prior to storage at −70 °C. Interleukin-6 (IL-6) ELISA (Quantikine^®^ ELISA, RnDSystems, Abingdon, UK) were carried out following the manufacturer’s guidelines and previously described [14]. To determine the pro-inflammatory status of the 3D organotypic models or NF following exposure to PGN and or Gap27 supernatants were subject to RnD systems Human Cytokine Array Panel A kits (RnD Systems, Abingdon, UK). Membranes were developed with streptavidin-horse radish peroxidase conjugate and detected via chemiluminescence using an Odyssey FC Dual Mode imaging system and LiCOR Image Studio software (LiCOR, Cambridge, UK). Data was calculated by dot intensity analysis and background levels subtracted to permit relative pixel intensity (RPI) to be extracted. Data are presented as the fold change in cytokine expression over control samples.

### 4.8. Statistical Analysis

All experiments were performed in triplicate and on at least three separate occasions from at least three different donors. All values indicate the mean ± SEM and the number of independent experiments (N); data was compiled and statistically analysed using appropriate tests for data sets (stated in figure legends) in GraphPad Prism 9 (GraphPad software, USA; www.graphpad.com (accessed on 20 May 2021)) (* *p* < 0.05; ** *p* < 0.005, *** *p* < 0.001).

## Supplementary Materials

Supplementary Materials can be found at https://www.mdpi.com/article/10.3390/ijms22116060/s1.

## Abbreviations

3D: three dimensional; ALI: air liquid interface; ATP: Adenosine tri phosphate; CCL5/RANTES: Chemokine ligand 5; CK16: cytokeratin16; CP: chronic plaque psoriasis; CX: connexin; CXCL-1: chemokine (C-X-C motif) ligand **1**; DAPI: 4′,6-diamidino-2-phenylindole; DMEM: Dulbecco’s Modified Eagle’s Medium; ERK: extracellular signal-regulated kinase; GAPDH: glyceraldehyde 3-phosphate dehydrogenase; GCSF: granulocyte colony-stimulating factor; GJ: Gap Junction; GMCSF: granulocyte-macrophage colony-stimulating factor; H&E: haemotoxylin and eosin staining; HC: hemichannel; HF: palmaplantar psoriasis; IL: interleukin; MAP: mitogen activated phosphorylation; MCP-1: monocyte chemoattractant protein 1; MFI: mean fluorescence intensity; MIF: macrophage migration inhibitory factor; NF: normal fibroblasts; NN: normal donor tissue; Panx1: pannexin 1; PASI: psoriasis associated severity index; PF: psoriatic fibroblasts; PGN: peptidoglycan; PKA: protein kinase A; PKC: protein kinase C; PN: non-involved psoriatic tissue; PP: psoriatic plaque tissue; Pser368: phosphorylated serine at position 368; RPI: relative pixel intensity; RT-PCR: real-time quantitative polymerase chain reaction; SEM: standard error of the mean; SP: scalp psoriasis; TLR: toll-like receptor; WGA: whole genome analysis.
