# Supplementary material for: Dysregulation of Connexin Expression Plays a Pivotal Role in Psoriasis
Source: Int J Mol Sci. 2021 Jun 4;22(11):6060. doi: 10.3390/ijms22116060 (PMC8200029; doi:10.3390/ijms22116060)
Supplement: Supplementary file 1 [file ijms-22-06060-s001.zip › ijms-1138595-supplementary.pdf]

Table S1. Primer sequences for Real-Time PCR.

Gene	Primers	Tm (°C)	Probe
Cx43F	ACT GGC GAC AGA AAC AAT TCT TC	57.1	CGC AAT TAC AAC AAG CAA GCA AGT GAG C
Cx43R	TTC TGC ACT GTA ATT AGC CCA GTT		
Cx26F	AAG CCA GTT TAA CGC ATT GCC CAG	62.3	TAG ACA GCA TGA GAG GGA TGA GGC AA
Cx26R	AAT GCT AGC GAC TGA GCC TTG ACA		
IL-6F	GCAGAAAACAACCTGAACCTT	54.9	TTCACCAGGCAAGTCTCCTCATTGAATCCAGA
IL-6R	ACCTCAAACTCCAAAAGACCA		
IL-17F	CCTCAGATTACTACAACCGATCC	56.1	CCACCGCAATGAGGACCCTGAGAGA
IL-17R	CACTTTGCCTCCCAGATCAC		
TLR2F	GCCGCAACTCAAAGAACTTTAT	55.1	TGATGACTCTACCAGATGCCTCCCTCTTACCC
TLR2R	GCATTCCTACTGATTTTCAATACTAG		
Cx30F	CTAACTTGTACGCCCTCTTCATG	56.8	AATATTCCAGGCGACATCCTCACTGACCG
Cx30R	GCACAGTGATGATACGAATGTCA		
Ki67F	TGT CGT CGT TTG TTT GCC TAT	55.9	AGT TGT TCC TGC CAC CGT GCC CTG
Ki67R	CTC ATC CAT TCA TTC GTG TTT ACC		
Panx1F	TGC TCA GTT CCA GGT GTT AC	56	AGG GCA AAG TTT GTG GGA GGT ATC TG
Panx1R	CGG CAG CTA ATG TAC TTG ATG		
GAPDHF	CAC ATG GCC TCC AAG GAG TAA	58.5	CTG GAC CAC CAG CCC CAG CAA G
GAPDHR	TGA GGG TCT CTC TCT TCC TCT TGT		
